# General Cognitive Impairment as a Risk Factor for Motor Vehicle Collision Involvement: A Prospective Population-Based Study

**DOI:** 10.3390/geriatrics3010011

**Published:** 2018-03-06

**Authors:** Carrie Huisingh, Cynthia Owsley, Virginia G. Wadley, Emily B. Levitan, Marguerite R. Irvin, Paul MacLennan, Gerald McGwin

**Affiliations:** 1 Department of Ophthalmology, School of Medicine, University of Alabama at Birmingham, Birmingham, AL 35294, USA; owlsey@uab.edu (C.O.); mcgwin@uab.edu (G.M.J.); 2 Division of Gerontology, Geriatrics, and Palliative Care, School of Medicine, University of Alabama at Birmingham, Birmingham, AL 35294, USA; vwadley@uab.edu; 3 Department of Epidemiology, School of Public Health, University of Alabama at Birmingham, Birmingham, AL 35294, USA; elevitan@uab.edu (E.B.L.); irvinr@uab.edu (M.R.I.); 4 Department of Surgery, School of Medicine, University of Alabama at Birmingham, Birmingham, AL 35294, USA; maclennan@uab.edu

**Keywords:** MMSE, crash, older drivers, cognitive decline

## Abstract

This study examined whether cognitive impairment and decline as assessed by a brief mental status screening test is associated with future crash risk in a cohort of older drivers. A three-year prospective study was conducted in a population-based sample of 2000 licensed drivers, aged 70 years and older. At the baseline visit, cognitive impairment was defined as <24 on the Mini Mental State Exam (MMSE). Decline was defined as those with a one-year change in MMSE scores in the lowest quartile (largest decrease). Motor vehicle collision involvement was obtained from the Alabama Department of Public Safety. Poisson regression was used to calculate crude and adjusted rate ratios (RR). There were 278 crashes during the follow-up period. Rates of crash involvement were higher for those with cognitive impairment (crude RR = 2.33) compared to those without impairment at baseline; adjustment for potential confounders namely age and visual processing speed attenuated this relationship (adjusted RR = 1.26, 95% confidence interval (CI) 0.65–2.44). Drivers who experienced a pronounced decline in estimated MMSE scores in one year were 1.64 (95% CI 1.04–2.57) times more likely to have a future at-fault crash, as compared to those whose scores did not decline. Evaluation of MMSE over time may provide important insight in an older driver’s future risk of at-fault crash involvement.

## 1. Introduction

In 2014, there were almost 40 million licensed drivers aged 65 and older in the United States, a 22% increase from 2004 [1,2]. Driving helps older adults stay mobile and independent, but the risk of being injured or killed in a motor vehicle crash (MVC) increases with age. Involvement in fatal crashes per mile traveled begins increasing among drivers aged 70–74 and is highest among those aged 85 and older [3]. Advancing age is also associated with a decline in functional capabilities that may affect driving skills. Of particular concern to clinicians and traffic safety researchers are impairments in cognitive function. Control of a vehicle places demand on attention, memory, and perception—cognitive skills that may decline with age [4]. Cognitive function in older adults is a continuum from normal to impaired, with varying severity of symptoms and underlying pathologies. Mild cognitive impairment (MCI), a concept between normal cognitive changes of aging and very early dementia, affects 10 to 20% of community-dwelling older adults 65 and older [5]; the annual rates of progression from MCI to dementia range from 5 to 20% in this age group [5]. Prevalence of dementia doubles with every five years of age over 65, such that prevalence estimates for adults over 90 reach as high as 50% [6]. Older drivers with cognitive impairment due to dementia are more than twice as likely to have been involved in a police-reported crash [7,8,9] and be at-fault [9]. While there is broad international consensus that those with a diagnosis of severe dementia should not drive or warrant driving restrictions [10], persons with MCI have more subtle decrements in driving skills [11]. It is unclear whether those with cognitive dysfunction that does not yet meet the diagnostic criteria for dementia are at an increased risk of crash involvement. 

The Mini-Mental State Exam (MMSE) is a general cognitive screening assessment widely used in both clinical and research settings [12]. The MMSE can be administered in 5–10 min and scores range from 0–30; scores less than 24 are often used to detect dementia. Studies on general cognitive function and driving abilities have employed samples derived from clinical settings such as primary care or specialty clinics [13,14,15,16,17,18,19], or have selected subjects for study based on a known diagnosis of dementia [7,20,21,22], or loss of license [23]. The results of studies based on convenience samples may be biased due to referral patterns and complicated by severity or duration of illness, or degree of functional impairment. Several studies have focused on driving performance using an on-road driving assessment or a driving simulator, showing that poor performance on the MMSE as well as other general cognitive tests such as the Montreal Cognitive Assessment, Trails B, Clinical Dementia Rating scale, and Short Blessed Test is associated with poor driving performance [9,13,18,19,20,21,24,25,26,27,28,29,30,31]. Driving performance is linked to driving safety theoretically, but this link is not well-established empirically [32]. Few studies have assessed motor vehicle crash risk, and the majority used self-reported crash involvement which is subject to recall bias [7,13,14,15,17,33,34,35].

To our knowledge, only two prospective population-based studies of older adults have examined the relationship between cognitive impairment as assessed by the MMSE and future crash risk using police-reported records. Rubin et al. reported a crude hazard ratio of 0.91 for a 1-point higher increment in MMSE score [36]. A study of older women by Margolis et al. reported no association [37]. Neither study used a clinically meaningful cut-off to define impairment, and both studies take into account MMSE scores only at a single time point but did not consider the changes in MMSE scores over time. It has been demonstrated that changes in MMSE are significantly associated with neurocognitive changes consistent with Alzheimer’s disease or other pathological processes or events [38]. Given that the MMSE is recommended in published guidelines as useful for identifying those at increased risk for unsafe driving [10], it is important to elucidate whether scores on the MMSE are associated with crash involvement, particularly in the population of drivers aged 70 and older rather than a clinic-based sample.

The purpose of this study was to examine whether impaired general cognitive function as assessed by the MMSE and 1-year change in MMSE scores are risk factors for crash involvement using a prospective population-based sample of older drivers.

## 2. Materials and Methods

### 2.1. Study Cohort

The study cohort consisted of a population-based sample of licensed drivers aged 70 years and older who resided in Jefferson County, Alabama or the bordering counties located in north-central Alabama [39]. Potential participants were identified from contact information available through a list of persons in this geographic area obtained from a direct marketing company (Pinpoint Technologies, Tustin, CA, USA). After confirming driver’s license status through the Alabama Department of Public Safety, potential participants were randomly selected from the final list and contacted by letter and followed-up with a phone call. Participants were enrolled between October 2008 and August 2011. Those who stated they had an Alabama license, had driven within the last three months, and spoke English were eligible to participate. The final sample consisted of 2000 drivers. Participants completed a single in-person visit at the Clinical Research Unit at the University of Alabama at Birmingham (UAB) and were followed-up with telephone calls at one-year intervals for three subsequent years. The study was approved by the Institutional Review Board at UAB and was consistent with the Declaration of Helsinki.

### 2.2. Data Collection

Following written informed consent, a trained interviewer administered the MMSE to assess general cognitive status [12]. The MMSE is a 30-item gross cognitive screening measure that assesses orientation to time and place, registration, memory, attention and concentration, and language. For the MMSE, scores below 24 of a possible 30 have been shown to detect dementia fairly accurately and non-demented older subjects usually score 24 or above [12]. For that reason, impaired cognitive status at baseline was defined as a MMSE score of <24. Because clinical guidelines suggest using an MMSE score of <25 to identify patients at increased risk for unsafe driving [10], this cut-off was used as well to see if results were consistent.

At the baseline visit, a demographic review (age, sex, race, education, and marital status), a general health questionnaire about the presence or absence of chronic medical conditions (i.e., “has a doctor ever told you that you have…”) [40], and questions about smoking and alcohol use were also administered. The baseline visit included a series of visual screening tests. Tests for central vision included visual acuity using the Electronic Visual Acuity (EVA) system [41], contrast sensitivity using the Pelli-Robson Contrast Sensitivity chart [42,43], and visual field sensitivity with the Humphrey Field Analyzer (HFA) Model II-I (Carl Zeiss Meditec, Dublin, CA, USA) using a 20-point custom test design. In addition, spatial ability as assessed by the Visual Closure Subtest of the Motor-free Visual Perception (MVPT) test [44] and visual processing speed as measured by the Trails B [45] and Useful Field of View (UFOV) subtest 2 (Visual Awareness Research Group, Punta Gorda, FL, USA) [46] were collected. A detailed description of the vision tests has been published previously [47]. For all testing, measurements were administered under binocular viewing using habitual correction, so participants wore whatever spectacles or contact lenses they normally wear when driving.

During the three annual follow-up phone calls, the abbreviated 6-item Orientation-Memory Concentration (OMC) test was administered. The OMC has been shown to reliably discriminate among mild, moderate and severe cognitive deficits [48] and has a strong established association with the MMSE. The OMC requires identifying the current year and month, identifying time within 1 h, counting backwards from 20 to 1, saying the months in reverse order, and repeating a name and address which the test administrator has told the subject earlier in the assessment. Weighted scores on the test range from 0 (no errors) to 28 (maximum errors). In order to investigate cognitive change over time, a previously published conversion algorithm was used to convert OMC scores to MMSE scores (MMSE = 32.0 – (0.75 × OMC)) [49]. This allowed us to calculate the change in function from baseline to year 1 using the same scale. Those with a decline in MMSE of ≥1 point fell in the lowest quartile of change in cognitive function and were classified as having cognitive decline.

At each telephone survey, the interview included questions about driving status and cessation. Driving cessation was defined as a negative response to the question, “Do you currently drive?” Participants who reported driving cessation were asked when they stopped driving. Five participants were excluded for inconsistent response regarding driving cessation (i.e., they reported being a current driver at baseline, but during follow-up reported a date of driving cessation prior to study entry). Therefore, baseline and telephone survey data from 1995 participants were included in the analysis. While it is possible for driving status to switch multiple times (e.g., current driver, then former driver, then back to current driver) over the study period, participants’ data were censored after the first report of driving cessation.

Information about participants’ MVC involvement occurring during the study period was made available by the Alabama Department of Public Safety. Of relevance, these reports provided the date of the collision and at-fault status according to the police officer at the scene. For each participant, the number of any and at-fault police-reported MVC subsequent to enrollment was the outcome of interest.

Date of death was confirmed by searching the Social Security Death Index or newspaper obituaries. Participants were considered at-risk for MVC involvement until the earliest occurrence of death, driving cessation, or three years after the participant’s enrollment date.

An estimate of driving exposure (e.g., miles driven in a typical week) was generated through the administration of the Driving Habits Questionnaire (DHQ) at baseline and at each telephone survey. The DHQ is a valid and reliable instrument for estimating driving exposure [50]. A structured interview that asked about the places driven to in a typical week as well as their distance from home was used to estimate the amount of driving done in a typical week. From each interview, an estimate of total annual mileage was computed. To calculate total mileage during the study, the mileage reported during each interview was summed while the participants were considered at-risk. If a participant was no longer at risk, the annual mileage was multiplied by the proportion of time they completed from the prior interview. For example, if a death occurred in July and the last interview occurred in January, the annual mileage reported in January was multiplied by 0.50.

### 2.3. Statistical Analysis

Demographic, visual, medical, driving characteristics were compared between those with and without cognitive impairment at baseline. These variables were compared using chi-square and *t*-tests for categorical and continuous variables, respectively. Poisson regression was used to calculate crude and adjusted rate ratios (RRs) and 95% confidence intervals (CI) to assess the association between cognitive status at baseline and rates of any and at-fault MVC involvement. The models used a log link function and accounted for driving exposure using the natural log of the annual miles driven using an offset term. Models were adjusted for age, gender, race, education, prior at-fault crash involvement, visual sensory function (i.e., visual acuity, contrast sensitivity, driving visual field), and visual-cognitive function (i.e., spatial ability, visual processing speed) because these are known confounders for the association between cognitive status and crash involvement.

To examine the association between cognitive decline and crash risk, only those crash events that occurred after the 1-year follow-up visit were included. This was done to ensure that the cognitive decline preceded any MVC involvement. The primary independent variable was modeled in two ways. First, cognitive change, operationalized as the change in MMSE score from baseline to year 1, was modeled as a continuous variable. Second, cognitive decline was modeled as a dichotomous variable to assess if rates of crash involvement were higher for those who declined ≥1 point on the MMSE (lowest quartile) compared to those who did not (upper three quartiles). For all statistical tests, a two-sided *p-*value < 0.05 was considered statistically significant.

## 3. Results

At baseline, 2.3% (*n* = 46) of the drivers had an MMSE score <24 and 4.3% (*n* = 86) had an MMSE score <25. Results were consistent with both definitions of cognitive impairment, so those based on MMSE scores <24 are presented. Compared to those who were not cognitively impaired, those with cognitive impairment were more likely to be older (mean age 81.0 vs. 77.1 years), non-white (45.7% vs. 17.4%), and have less than a high school education (50.0% vs. 31.2%) (Table 1). There were no differences according to sex, number of falls in the past year, number of medical conditions, or presence of any chronic medical conditions. By design, those with cognitive impairment had lower MMSE scores compared to those without impairment (mean score 20.8 vs. 28.4). Those with cognitive impairment were also more likely to have impaired visual acuity, contrast sensitivity, lower visual field sensitivity, and impaired visual processing speed as measured by the UFOV and Trails B. The number of miles driven in the previous year was lower among those with cognitive impairment compared to those without cognitive impairment; however, this difference was not statistically significant. Those with cognitive impairment at baseline were more likely to report having prior crash and at-fault crash involvement in the previous 5 years.

During the follow-up period, there were 249 participants involved in 278 MVCs. Overall, rates of crash (crude RR = 2.33) and at-fault crash (crude RR = 3.45) involvement per mile driven were significantly higher for those with cognitive impairment compared to those without cognitive impairment at baseline (Table 2). However, after adjusting for potential confounders, the association was attenuated and the associations for crash involvement (adjusted RR = 1.26, 95% CI 0.65–2.44) and at-fault crash involvement (adjusted RR = 1.37, 95% CI 0.60–3.11) were not significantly different between those with and without cognitive impairment at baseline. The main variables attenuating the relationship were age and visual processing speed impairment status as measured by the UFOV and Trails B.

There were 1937 who participated in the year 1 follow-up interview. Compared to those who did not contribute, participants were younger, had higher baseline MMSE scores, and were more likely to have impaired visual acuity, visual field sensitivity, and MVPT and slowed visual processing speed (*p-*value < 0.05 for all). Between baseline and year 1, the sample had a mean increase of 0.51 (SD 2.78) on the MMSE, ranging from a decline of −13.8 to an increase of 10.0 points. One hundred seventy-seven crash events occurred after the 1-year follow-up visit and were included in this analysis (Table 3). The quadratic term did not indicate the linearity assumption between MMSE score as a continuous measure and any and at-fault crash involvement was significantly different from zero. Therefore, the interaction term was removed and change in MMSE was treated as a continuous variable in the model. Change in MMSE as a continuous variable was not associated with crash or at-fault crash involvement in the crude or adjusted models. Compared to those in the upper three quartiles, rates of at-fault crash involvement were 1.73 times higher for those who declined ≥1 point between baseline and the year 1; after adjustment, the association persisted (adjusted RR = 1.64, 95% CI 1.04–2.57). Rate ratios were not significantly altered even with additional adjustment for the baseline MMSE score (adjusted RR = 1.69, 95% CI 1.08–2.66).

## 4. Discussion

As the U.S. population ages, the number of drivers with cognitive impairment is expected to increase. The MMSE is widely regarded as a valid and reliable instrument for assessing cognitive impairment [51]. Lower scores on the MMSE as well as other general cognitive screening tests have been associated with a greater likelihood of poor driving performance and motor vehicle collision involvement, though not consistently. In this prospective cohort study of older drivers, the prevalence of cognitive impairment using a standard screening threshold for possible dementia was only 2.3%. Those with cognitive impairment at baseline had a higher crash rate in the crude analysis, but did not have significantly higher rates of crash or at-fault crash involvement per mile driven in the subsequent 3 years after adjusting for potential confounders. Between baseline and year 1, the 25% of participants who declined ≥1 point on the estimated MMSE had significantly higher rates of at-fault crash involvement over the next two years compared to those who did not decline.

Not surprisingly, the prevalence of cognitive impairment observed in the current study was low compared to other population-based reports. For example, in a subsample drawn from the Reasons for Geographic and Racial Differences in Stroke (REGARDS) study of older Black and White adults, the prevalence of cognitive impairment based on the Six Item Screener was 5.1% [52]. However, the present study uses a fundamentally different population. In the present study, participants were randomly selected from a list of licensed drivers. Therefore, this cohort does not represent the entire older adult population, but rather those who are licensed to drive and are current drivers. This suggests, that while there are some cognitively impaired drivers on the road, the prevalence of cognitive impairment among current drivers is relatively low. Lower MMSE scores are associated with driving cessation [53,54], so it is likely that many individuals with dementia-level cognitive impairment were no longer current drivers and thus were not eligible for enrollment.

A baseline MMSE score of <24 was not associated with future crash involvement after adjusting for potential confounding factors. This finding is consistent with other prospective studies [35,37]. Some driving guidelines suggest a total MMSE score of <25 is a better indicator of crash risk [10]; however, an MMSE score based on this cut-off was also not associated with future crash risk in our analyses. The lack of association may be because the MMSE focuses more on verbal cognitive skills and has less emphasis on skills that are more closely related to driving ability [4,55]. Previous population-based research suggests that visual attention, visual search (processing speed), and spatial understanding and memory are associated with crash involvement [27,34,40,56,57]. In fact, using the same sample of drivers as the current study, performance-based measures, such as the Trails B that test cognitive and functional skills necessary for safe driving, were strongly associated with future crash risk even after adjusting for mental status [57,58]. Adjusting for the performance-based measures attenuated the association between the MMSE and crash rates in this study. Still, specific cognitive domains of the MMSE have been associated with other types of mobility in older adults such as falling [59], hip fracture [60], rehabilitation outcomes [61], and even crash involvement [62]. Future studies are needed to examine whether the specific areas of cognitive functioning assessed by the MMSE score are related to crash risk.

Our finding that participants tested one year after baseline gained approximately +0.5 points on the MMSE score is likely due to several factors. First, the mode of administration differed since the MMSE was done in person and the OMC was done over the phone, so the mode of administration may have added a source of variability to the scores. In addition, exposure to testing in general may be expected to produce small practice effects [63]. Finally, the selective attrition of lower functioning participants, which is a ubiquitous phenomenon in longitudinal research, likely underestimated the extent of decline in the full sample [64]. In terms of the magnitude of change, a previous study reported a one-year decline of −0.9 MMSE points, but was based on a clinical sample with high baseline rates of cognitive impairment, which differs substantially from our community-based sample of older drivers [65]. That said, a gain of 0.5 points in the current study is equivalent to 0.18 SDs of the distribution. This change is below the 0.50 SD threshold considered to represent clinically meaningful change [66].

Those with substantial cognitive decline between baseline and year 1 had higher rates of at-fault crash involvement, not all-cause crash involvement. From an etiologic perspective, this is interesting because at-fault crashes are more likely to be tied to the drivers’ functional characteristics, in this case, their cognitive function. Drivers who declined in function were more likely to be older, non-White, have lower educational attainment, and impaired visual acuity and visual-cognitive functioning (Table 4). Even after adjusting for these differences, the association with at-fault crash risk persisted. Cognitive decline is associated with other outcomes such as changes in functioning related to activities of daily living, concentration and memory [65], reduced mobility, loss of autonomy, and shorter lifespan [67]. It is possible that those with a substantial decline in MMSE have a more aggressive course of underlying neurological disease with a worse prognosis, which could influence their functional capacity to safely drive.

Clinicians are often asked to make a recommendation about the need to limit or stop driving for their elderly patients. General cognitive assessments such as the MMSE are often used in the clinic and may inform their recommendation on driving safety. The results of this study suggest that clinicians should be encouraged to educate patients about driving safety when patients experience a substantial decline in cognitive function (i.e., 1 or more points) in 1 year. Our results show that the association between MMSE and crash risk was confounded by other risk factors, particularly performance-based measures of visual-cognitive functioning. Nevertheless, the MMSE is well integrated into primary care, so this indirect relationship may still serve to identify a high-risk group of drivers and is therefore useful for public health purposes. In addition, more research is needed to understand the long-term care and transportation needs of those who experience a substantial cognitive decline.

Our study has several strengths and limitations worth noting. This was a large population-based sample of older drivers who were unselected for dementia and followed prospectively for MVC involvement, which was based on police-reported crashes and is considered the gold standard. Participants self-identified as being current drivers, which allowed us to exclude those who were licensed for identification purposes but did not drive. Models accounted for miles driven. Information on several important factors known to affect crash risk were included and adjusted for in this analysis; however, it is possible that variables not adjusted for may have affected the results such as depression [68]. This cohort did not exclude subjects with a MMSE score below a certain threshold and allowed us to identify older drivers with cognitive impairment; therefore, results are generalizable to community-dwelling licensed older drivers, not those seen in a tertiary care or referral clinic. While cognitive assessments were conducted over time, the MMSE was implemented at the baseline assessment whereas the OMC was done at the annual intervals. This was done because the MMSE includes a copy design task which requires the participant to complete the task in front of the interviewer. In contrast, the OMC is designed for use over telephone. Nevertheless, an established conversion algorithm was used to convert OMC scores to the MMSE. Results should be replicated using the same assessment tool to rule out any unmeasured differences by mode of assessment. When examining cognitive decline, any crash that preceded the year 1 assessment was excluded from the analysis. This was done to ensure that the cognitive decline preceded any MVC involvement and to ensure that cognitive decline was not affected by these events. This led to a trade-off between a shorter follow-up period to calculate cognitive decline while maintaining a large enough number of crash events that could still generate meaningful conclusions. However, it would be valuable to calculate the rate of MMSE change occurring over a longer time period while using a longer period of observing crash events. This should be addressed in future studies.

## 5. Conclusions

In conclusion, this study indicates that substantial cognitive decline occurring in 25% of adults 70 and older elevates the rate of at-fault MVCs, even after accounting for potentially confounding factors. With the aging of the U.S. population and increasing prevalence of older adults who drive with cognitive impairment, evaluation of MMSE over time may provide important insight into their future risk of at-fault crash involvement.

## Figures and Tables

**Table 1:** Demographic, visual, medical, and driving characteristics by mental status (Mini Mental State Exam (MMSE)) at baseline (*N =* 1995).

Characteristics	Impaired ^1^ *n =* 46	Not Impaired *n =* 1949	*p-*Value
Age, years			
70–79	20 (43.5%)	1410 (72.4%)	<0.0001
80–89	24 (52.2%)	501 (25.7%)	
90–98	2 (4.4%)	38 (2.0%)	
Mean, SD	81.0 (±6.0)	77.1 (±4.9)	<0.0001
Gender			
Male	31 (67.4%)	1092 (56.0%)	0.12
Female	15 (32.6%)	857 (44.0%)	
Race			
Non-White	21 (45.7%)	340 (17.4%)	<0.0001
White	25 (54.4%)	1610 (82.6%)	
Education			
Less than high school	23 (50.0%)	608 (31.2%)	0.0008
High school or GED	4 (8.7%)	47 (2.4%)	
1–4 years of college	13 (28.3%)	1001 (51.4%)	
Postgraduate degree	6 (13.0%)	292 (15.0%)	
Falls in past year			
0	36 (78.3%)	1496 (76.8%)	0.96
1	6 (13.0%)	284 (14.6%)	
≥2	4 (8.7%)	169 (8.7%)	
Number of medical conditions			
0	2 (4.4%)	80 (4.1%)	0.30
1–2	20 (43.5%)	617 (31.7%)	
3–4	18 (39.1%)	830 (42.6%)	
5 or more	6 (13.0%)	422 (21.7%)	
Chronic medical conditions			
Heart problems	19 (41.3%)	776 (39.8%)	0.84
Circulation problems	4 (8.7%)	314 (16.1%)	0.17
High blood pressure	25 (54.4%)	1281 (65.7%)	0.11
Low blood pressure	3 (6.5%)	103 (5.3%)	0.71
Neurological problems	4 (8.7%)	201 (10.3%)	0.72
Arthritis	27 (58.7%)	1064 (54.6%)	0.58
Osteoporosis	2 (4.4%)	275 (14.1%)	0.058
Cancer	8 (17.4%)	618 (31.7%)	0.039
Chronic pulmonary problems	7 (15.2%)	330 (16.9%)	0.76
Digestive problems	8 (17.4%)	556 (28.5%)	0.097
Urinary problems	17 (37.0%)	620 (31.8%)	0.46
Kidney problems	4 (8.7%)	176 (9.0%)	0.94
Hearing problems	21 (45.7%)	627 (32.2%)	0.054
MMSE, mean (SD)	20.8 (±2.8)	28.4 (±1.5)	<0.0001
Min-Max	10–23	24–30	---
Visual acuity, logMAR (OU)			
≤0.0 (not impaired)	33 (73.3%)	1799 (92.4%)	<0.0001
>0.0 (impaired)	12 (26.7%)	149 (7.7%)	
Contrast sensitivity, log sensitivity (OU)			
<1.5 (impaired)	7 (15.2%)	123 (6.3%)	0.016
≥1.5 (not impaired)	39 (84.8%)	1825 (93.7%)	
Overall visual field sensitivity (dB)			
≤22.5 (worse)	28 (60.9%)	465 (23.9%)	<0.0001
>22.5 (better)	18 (39.1%)	1484 (76.1%)	
Visual processing speed, UFOV test (ms)			
<150 (not impaired)	0 (0.0%)	1125 (57.8%)	<0.0001
150–350	16 (34.8%)	636 (32.7%)	
>350 (impaired)	30 (65.2%)	187 (9.6%)	
Visual processing speed, Trails B, minutes			
<2.47 (not impaired)	1 (2.2%)	1240 (63.8%)	<0.0001
≥2.47 (impaired)	45 (97.8%)	704 (36.2%)	
Motor-Free Visual Perception Test, # correct			
<8 (impaired)	28 (60.9%)	270 (13.9%)	<0.0001
≥8 (not impaired)	18 (39.1%)	1679 (86.2%)	
Annual mileage, prior year	7611 (±10,113)	9579 (±9412)	0.16
No. of MVCs in prior 5 years			
0	27 (58.7%)	1434 (73.6%)	0.024
1 or more	19 (41.3%)	515 (26.4%)	
No. of at-fault MVCs in prior 5 years			
0	33 (71.7%)	1696 (87.0%)	0.0026
1 or more	13 (28.3%)	253 (13.0%)	

^1^ Impairment defined as MMSE score <24. Numbers are means ± SD or n (%). Chi-square tests and *t*-tests were used to calculate *p-*values for categorical and continuous variables, respectively. Abbreviations: dB, decibels; logMAR, log minimum angle resolvable; MMSE, mini-mental state examination; ms, milliseconds; SD, standard deviation; UFOV; useful field of view.

**Table 2:** Crude and adjusted association between cognitive impairment status at baseline and rate of future motor vehicle crash (MVC) involvement (*N* = 1995).

Cognitive Impairment Status	No. of Drivers	Any MVC					At-Fault MVC				
		No. of Crashes	Crude	Adjusted ^2^			No. of Crashes	Crude	Adjusted ^2^		
			RR	RR	95% CI	*p-*Value		RR	RR	95% CI	*p-*Value
Impaired ^1^	46	11	2.33	1.26	0.65–2.44	0.50	8	3.45	1.37	0.60–3.11	0.46
Not impaired (ref)	1949	267	1.0	1.0	---		131	1.0	1.0	---	

^1^ Impairment defined as MMSE score <24; ^2^ Adjusted for age, gender, race, education, prior at-fault crash involvement, and visual acuity, contrast sensitivity, visual field, UFOV, Trails B, and MVPT impairment status. An offset term was used to adjust for annual mileage. Abbreviations: CI, confidence interval; MMSE, mini-mental state examination; ref, reference; RR, rate ratio; *N* = 5 participants reported a driving cessation date prior to study enrollment despite being a current driver at enrollment and were excluded from this analysis.

**Table 3:** Crude and adjusted association between cognitive decline from baseline to one-year follow-up and rate of future MVC involvement (*N* = 1937).

Cognitive Decline	No. of Drivers	Any MVC					At-Fault MVC				
		No. of Crashes	Crude	Adjusted ^3^			No. of Crashes	Crude	Adjusted ^3^		
			RR	RR	95% CI	*p-*Value		RR	RR	95% CI	*p-*Value
Change in MMSE ^1^ (continuous)	1937	177	0.98	0.99	0.94–1.04	0.67	86	0.93	0.94	0.87–1.01	0.098
Cognitive Decline ^2^											
Decline (∆ −13.75 to −1.0)	553	50	1.08	1.03	0.73–1.43	0.88	33	1.73	1.64	1.04–2.57	0.032
No decline (ref) (∆ −0.75 to +10.0)	1384	126	1.0	1.0	---		52	1.0	1.0	---	

^1^ Change defined as the difference in MMSE between baseline and year 1 follow-up (i.e., year 1 follow-up score minus baseline score); ^2^ Lowest quartile used as cut-off (−1.0) to define decline; ^3^ Adjusted for age, gender, race, education, prior at-fault crash involvement, and visual acuity, contrast sensitivity, visual field, UFOV, Trails B, and MVPT impairment status. An offset term was used to adjust for annual mileage. Abbreviations: CI, confidence interval; MMSE, mini-mental state examination; ref, reference; RR, rate ratio.

**Table 4:** Baseline characteristics of drivers with or without decline in function (*N* = 1937).

Characteristics	Decline (*n* = 553)	No Decline (*n* = 1384)	*p-*Value
Age, years			
70–79	372 (67.3%)	1024 (74.0%)	0.0043
80–89	166 (30.0%)	341 (24.6%)	
90–98	15 (2.7%)	19 (1.4%)	
Mean, SD	77.7 (±5.1)	76.9 (±4.8)	0.0015
Gender			
Male	324 (58.6%)	762 (55.1%)	0.16
Female	229 (41.4%)	622 (44.9%)	
Race			
Non-White	126 (22.8%)	220 (15.9%)	0.0004
White	427 (77.2%)	1164 (84.1%)	
Education			
Less than high school	203 (36.8%)	405 (29.3%)	0.0004
High school or GED	21 (3.8%)	27 (2.0%)	
1–4 years of college	249 (45.1%)	736 (53.2%)	
Postgraduate degree	79 (14.3%)	216 (15.6%)	
Falls in past year			
0	429 (77.6%)	1065 (77.0%)	0.26
1	71 (12.8%)	209 (15.1%)	
≥2	53 (9.6%)	110 (8.0%)	
Number of medical conditions			
0	20 (3.6%)	61 (4.4%)	0.71
1–2	178 (32.2%)	443 (32.0%)	
3–4	232 (42.0%)	598 (43.2%)	
5 or more	123 (22.2%)	282 (20.4%)	
Chronic medical conditions			
Heart problems	215 (38.9%)	553 (40.0%)	0.66
Circulation problems	91 (16.5%)	209 (15.1%)	0.46
High blood pressure	368 (66.6%)	894 (64.6%)	0.42
Low blood pressure	24 (4.3%)	79 (5.7%)	0.23
Neurological problems	65 (11.8%)	128 (9.3%)	0.096
Arthritis	297 (53.7%)	760 (54.9%)	0.63
Osteoporosis	69 (12.5%)	200 (14.5%)	0.26
Cancer	187 (33.8%)	420 (30.4%)	0.14
Chronic pulmonary problems	96 (17.4%)	222 (16.0%)	0.48
Digestive problems	160 (28.9%)	388 (28.0%)	0.69
Urinary problems	170 (30.7%)	443 (32.0%)	0.59
Kidney problems	49 (8.9%)	122 (8.8%)	0.97
Hearing problems	166 (30.0%)	461 (33.3%)	0.16
MMSE, mean (SD)	28.2 (±1.8)	28.2 (±1.9)	0.56
Min-Max	21–30	10–30	
Visual acuity, logMAR (OU)			
≤0.0 (not impaired)	521 (94.2%)	1265 (91.5%)	0.046
>0.0 (impaired)	32 (5.8%)	117 (8.5%)	
Contrast sensitivity, log sensitivity (OU)			
<1.5 (impaired)	32 (5.8%)	91 (6.6%)	0.52
≥1.5 (not impaired)	521 (94.2%)	1292 (93.4%)	
Overall visual field sensitivity (dB)			
≤22.5 (worse)	147 (26.6%)	321 (23.2%)	0.12
>22.5 (better)	406 (73.4%)	1063 (76.8%)	
Visual processing speed, UFOV test (ms)			
<150 (not impaired)	275 (49.7%)	829 (59.9%)	<0.0001
150–350	193 (34.9%)	433 (31.3%)	
>350 (impaired)	85 (15.4%)	121 (8.8%)	
Visual processing speed, Trails B, minutes			
<2.47 (not impaired)	295 (53.4%)	923 (66.9%)	<0.0001
≥2.47 (impaired)	258 (46.7%)	456 (33.1%)	
Motor-Free Visual Perception Test, # correct			
<8 (impaired)	105 (19.0%)	179 (12.9%)	0.0007
≥8 (not impaired)	448 (81.0%)	1205 (87.1%)	
Annual mileage, prior year	9148 (±8631)	9653 (±9514)	0.28
No. of MVCs in prior 5 years			
0	403 (72.9%)	1018 (73.6%)	0.76
1 or more	150 (27.1%)	366 (26.5%)	
No. of at-fault MVCs in prior 5 years			
0	481 (87.0%)	1199 (86.6%)	0.84
1 or more	72 (13.0%)	185 (13.4%)	
